# Functional differentiation in pollination processes among floral traits in *Serapias* species (Orchidaceae)

**DOI:** 10.1002/ece3.3264

**Published:** 2017-08-02

**Authors:** Giuseppe Pellegrino, Francesca Bellusci, Anna Maria Palermo

**Affiliations:** ^1^ Department of Biology, Ecology and Earth Sciences University of Calabria Rende CS Italy

**Keywords:** floral display, male and female success, orchid, phenotypic selection, pollinaria, pollination strategy

## Abstract

Floral displays, influencing attractiveness to insects, increase the number of pollinator visits and the efficiency of each visit in terms of pollen exchange and thus affect the plant reproductive success. Here, we conducted an in situ manipulation experiment to investigate whether the floral modifications affect reproductive success in natural orchid populations of *Serapias lingua* and *Serapias vomeracea*. We estimated male and female reproductive success of three treatment groups, disassembly of floral tube, cutting of lip, and painting of the callus surface, in terms of pollinaria removed/deposited and fruit production. Results revealed that phenotypic modification had opposite effects on reproductive success of two examine species. Indeed, reproductive success was significantly increased by the detached of the petals and sepals, and decreased, due to callus painting and lip removal, in *S. lingua*. On the contrary, unmanipulated plants of *S. vomeracea* showed significantly higher value of pollinaria removed and deposited and fruit set than manipulated ones. The differences between *S. lingua* and *S. vomeracea* agree to the different pollination strategy of examined species. *S. vomeracea* shows shelter imitation strategy, and thus, the disassembly of tunnel‐like corolla does not allow the insects to use the flower as a refuge, while *S. lingua* is a sexually deceptive orchid and therefore the opening of the flower made more visible callus (visible at a greater distance) increasing the pollinators attraction. This study provides evidence that pollinators were largely sensitive to the experimental modification of the flower phenotype, which is consistent with the presence of significant selection on individual floral characters. Our experimental investigations of the effects of variation in display on pollinator visitation provide insights into the evolution of floral morphology in orchid with shelter imitation strategy.

## 1 INTRODUCTION

Flower morphology is known for their remarkable stability among individuals of the same species (Minelli, 2015). Flower structure may be a result of stabilizing selection imposed by local pollinator (Gómez, Perfectti, & Klingenberg, 2014; Parachnowitsch & Kessler, 2010). Phenotypic selection in natural populations and biotic interactions are important elements of the selection regime. Among biotic factors, pollinator behavior and preferences have been subject to manipulations in several studies of phenotypic selection (Sletvold, Grindeland, & Ågren, 2013).

The evolution of floral characters, above all traits involved in pollinator attraction, is under pollinator‐mediated selection in many plant species (Harder & Barrett, 2006). Color, size, number, and shape of flower or inflorescence have been demonstrated to impact pollinator attraction. Indeed, floral displays influencing attractiveness to pollinators (Biernaskie & Cartar, 2004) increase the number of visits received and the efficiency of each visit in terms of pollen exchange (Essenberg, 2012; Makino & Sakai, 2007). Many studies pointed out the relationship between floral corolla size, floral number, and pollinator attraction, suggesting that increased flower number or size increases visitation rates (Armbruster, 2014; Kaczorowski, Seliger, Gaskett, Wigsten, & Raguso, 2012; Zhao & Wang, 2015). A large number of studies have examined the effects of floral color, scent, and nectar on pollinator attraction, using both artificial and natural flowers (Dötterl, Glück, Jürgens, Woodring, & Aas, 2014; Kunze & Gumbert, 2001).

Manipulation of the floral phenotype has long been a part of studies of the mutualistic interaction between flowering plants and animal pollinators at the level of entire communities (Clements & Long, 1923; Mitchell, Irwin, Flanagan, & Karron, 2009; Moldenke, 1975). These studies have successfully demonstrated that pollinators can mediate selection on floral design associated with attraction and nectar reward as well as inflorescence design.

Orchids are highly represented in studies of floral evolution, due to their diverse and specialized pollination mechanisms (Jersáková, Johnson, & Kindlmann, 2006). Orchids have evolved an unparalleled diversity of floral visual signals, such as perianth color and form, and olfactory cues for pollinator attraction exploiting the innate sensory preferences of insects (Stökl, Brodmann, Dafni, Ayasse, & Hansson, 2011).

To demonstrate that a given floral trait causes differences in pollinator attraction, experimental manipulation of the trait is necessary. Trait manipulation experiments on orchids have demonstrated the reduction of fitness due to the shortening of nectar spurs (Boberg et al., 2014; Johnson & Steiner, 1997) and the influence of plant height on pollinator attraction (Sletvold, Grindeland, & Ågren, 2010). While modification of flower size may have different effects increasing or decreasing reproductive success (Boberg & Ågren, 2009; Pellegrino, Bellusci, & Musacchio, 2010). Approximately one‐third of the 25,000 described orchid species are deceptive (Dressler, 1990). Most of them are food deceptive species which attract foraging insects by mimicking the general signals employed by rewarding species (generalized food deception) or the specific signals of a co‐occurring rewarding model (food‐deceptive floral mimicry) (Jersáková et al., 2006 and reference therein). Several studies showed that visual signals than olfactory cues are the major ones involved in pollinator attraction in both types of food‐deceptive strategies (Galizia et al., 2005). Another remarkable deceptive mechanism of pollination is sexual deception of male bees and wasps (Kullenberg, 1961). Classical examples are those of sexually deceptive genus *Ophrys* that shows a close morphological and olfactory resemblance between mimic and model species (Tang, Ou, Luo, Zhuang, & Liu, 2014). Sexually deceptive orchids mimic signals emitted by female insects in order to attract mate‐searching males. The olfactory compounds produced by the labellum of the orchids act as long‐range attractants, guiding males to the proximity of flowers. At close range, also in sexually deceptive orchids, floral visual signal plays a key role in pollinator attraction and thus affects reproductive success (Rakosy, Streinzer, Paulus, & Spaethe, 2012).

In the last years, the pollination strategy of *Serapias* genus has received more attention, pointing out that *Serapias* species show two different pollination strategies. Indeed, *Serapias lingua* seems to have evolved sexually deceive pollinators, analogous to what is observed in *Ophrys* (Vereecken, Dafni, & Cozzolino, 2010).

This phenomenon is also supported by the finding of large amounts of alkanes and alkenes in its floral odor extracts (Pellegrino, Luca, Bellusci, & Musacchio, 2012; Schiestl & Cozzolino, 2008), and *Ceratina cucurbitina* males are its main pollinators (van der Cingel, 2001; Paulus, 2014; Vereecken et al., 2010). While an unusual type of deceptive pollination mechanism has been observed in the other *Serapias* species called shelter imitation strategy (Jersáková et al., 2006). In these cases, the sepals, petals, and lateral lobes of the hypochile form a tunnel‐like corolla, varying in diameter and depth among taxa, offering a floral tube to the insects in which they use to rest or sleep under bad or rainy weather conditions (van der Cingel, 2001).

Here, we assess the potential role of pollinator‐mediated selection on floral morphology through female and male reproductive success using experimental manipulations of floral traits. To examine the functions of attractive structures that differ in shape, it is necessary to investigate stages of the pollination process in relation to each attractive structure. We focus on three floral traits, broadly related to pollinator attraction: the form of flowers, the color of callosity at the base of the hypochile, and the presence of the lip. We manipulated the form of the *Serapias* flowers, transforming them from tubular form to open flowers, eliminating the lip and coloring the callosity, to examine the effects of such changes in attracting pollinators, and to quantify the effects on male and female reproductive success, that is, pollen removal and fruit/seed set, respectively.

**Figure 1:**
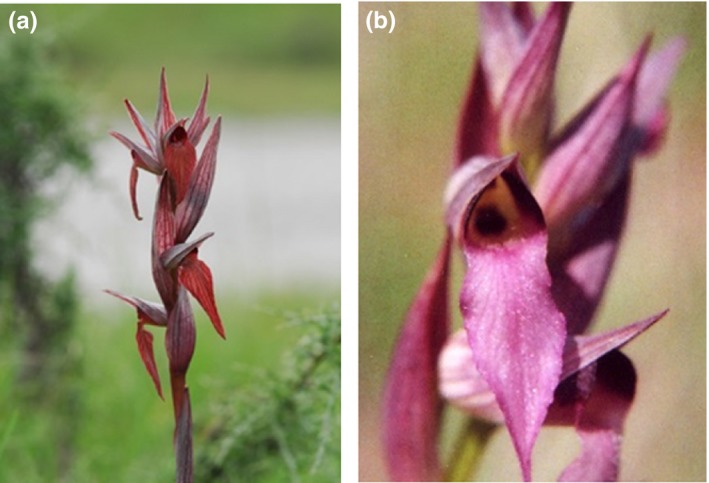
Photographs of *Serapias vomeracea* (a) and *Serapias lingua* (b)

Therefore, the aims of this study were to evaluate the effects of floral morphology and the role of the lip and the callus on pollination activity and to assess (1) if pollinators increase the rate at which they visit the flowers, as a result of responding to a more attractive “visual signal,” or (2) pollinators decrease their visits to modified flowers which are less attractive than the natural ones. We carried out flower manipulation experiments in four natural populations of *Serapias vomeracea* (Figure 1a) and *S. lingua* (Figure 1b) in Calabria (Italy) and measured male and female reproductive success over an 8‐week flowering period.

## 2 MATERIALS AND METHODS

### 2.1 Natural history

The experiments were conducted in April–June 2016 using *S. vomeracea* and *S. lingua* plants growing in four sympatric large wild populations located on an abandoned agricultural calcareous soil in the immediate vicinity of the National Park of Pollino (Calabria, Southern Italy). To minimize the effects of soil and vegetation types on measurements, we chose sites of matched vegetation types. All sites consist of calcareous, dry grasslands (Festuco‐Brometalia); *Spartium junceum* L., *Cytisus sessilifolius* L., and *Cistus incanus* L. are the frequent shrubs and *Festuca circummediterranea* Patzke, *Bromus erectus* Huds. and *Dactylis glomerata* L. are the dominant herbs.

The genus *Serapias* L. is distributed throughout the Mediterranean region with its centre of diversity in Southern Italy and on the Greek islands (Baumann & Künkele, 1989). A more recent systematic treatment included 30 taxa (Delforge, 2006), which were characterized by a common floral morphology, the lateral petals, and hypochile (the proximal part of the lip) form a hood (tubular corolla). Recent molecular analysis strongly supports a natural split of *S. vomeracea* and *S. lingua* into two different groups strictly related to two pairs of endemic species, *S. apulica* and *S. neglecta*,* S. gregaria* and *S. olbia*, respectively (Bellusci, Pellegrino, Palermo, & Musacchio, 2008).

*Serapias vomeracea* (Burm.) Briq. is a widespread species that grows in arid meadows, abandoned agricultural soils, garigues, and bushy environments up to 1000 m asl. It has a three‐lobed lip without a spur, a plain‐colored epichile more than 13 mm long and two guiding swellings at the base of the lip. The chromosome number is 2n = 36 (D'Emerico, Pignone, & Scrugli, 2000).

*Serapias lingua* (tongue orchid) is a short‐lived tuberous orchid and a tetraploid species (D'Emerico et al., 2000). It has dull‐colored flowers of uniform structure, with a callosity at the base of the hypochile, and the epichile (the distal part of the lip) generally inclined downwards. Conical epidermal papillae and two types of trichome with secretory apical cells characterize the petals and lip (Barone Lumaga et al., 2012). It grows in arid meadows, abandoned agricultural soils, garigue, and bushy environments up to 1,200 m a.s.l. (Delforge, 2006).

Both species are widespread species, co‐occur in the same habitat, mainly distributed in the Mediterranean‐Atlantic countries (Portugal, Spain, France, Italy, Balkans, Greece), but reaching western North Africa (Morocco, Tunisia), and show high level of inbreeding depression (Bellusci, Pellegrino, & Musacchio, 2009).

The pollination biology of *Serapias* orchids (included *S. vomeracea*) in the Mediterranean is usually viewed as a generalized mimicry of nests and shelter. Indeed, a wide range of insect pollinators, both males and females, were found immobile into the tubular flowers overnight with pollinaria attached to their head (Pellegrino, Gargano, Noce, & Musacchio, 2005; Pellegrino, Noce, Bellusci, & Musacchio, 2006). Moreover, the dark‐colored flowers accumulate warmth via the sun beams at dawn, providing the pollinators with a morning dose of heat presumably sufficient for them to start foraging earlier than others kept at a lower ambient temperature (Dafni, Ivri, & Brantjes, 1981). At least one species in the genus *Serapias*, namely *S. lingua*, seems to have evolved to sexually deceive pollinators, analogous to what is observed in *Ophrys* orchids (Jersáková et al., 2006). Moreover, preliminary observations indicate that *C. cucurbitina* males are the main pollinators (van der Cingel, 2001; Vereecken et al., 2010, 2012).

### 2.2 Manipulative experiments

**Figure 2:**
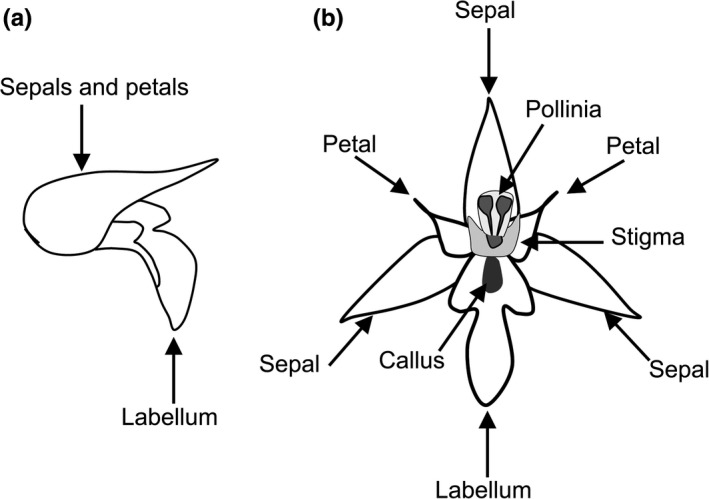
Drawing of a whole, with petals, sepals, and hypochile (the proximal part of the lip) forming a hood (a) and partitioned (b) flower of a *Serapias* species

To ensure that potential selection on flower traits occurred in relation to floral function, we modified traits beyond the range naturally observed in the population. In this way, we tested experimentally whether the floral traits under scrutiny represent relevant characters for pollinator attraction so that we can confidently ascribe adaptive function under putative selection. We focused on floral tube structure, labellum, and callus as these floral elements are what pollinators see when approaching flowers (Figure 2). To examine the effects of flower traits on pollination success and seed production, we performed three different types of manipulation at least on 15 individuals for each species and for each population. Therefore, the three manipulation experiments are as follows: (1) OPN, the petals, and sepals have been detached, and the tube shape was opened; (2) LAB, lip was completely removed with scissors; (3) CAL, the flower was open and white painting was applied to the callus surface. To verify whether the number of flowers can affect experiment results, these three treatments were performed on all flowers (‐100), on half flowers (‐50), and on 10% of flowers (‐10) of an inflorescence, and unmanipulated flowers were cut. In addition, at least 45 plants for each species and for each population were left unmanipulated and used as control (CON). To prevent natural pollination, in April 2016, approx. 200 individuals for each of the four sympatric populations for a total of approx. 440 plants for each examined species with flower buds were randomly chosen, individually tagged, and bagged. When the bagged flowers opened, we conducted one of the three treatments. Then, flowers were unbagged to allow pollination. Plant height (one‐way ANOVA; *F*_3, 368_ = 0.41, *p* = .64, *S. vomeracea*;* F*_3, 372_ = 0.38, *p* = .58, *S. lingua*), and number of flowers prior to experimental manipulation (one‐way ANOVA; *F*_3, 368_ = 0.34, *p* = .72, *S. vomeracea*;* F*_3, 372_ = 0.35, *p* = .68, *S. lingua*) did not differ between the four (three manipulations + control) treatment groups.

Pollinaria removal and pollen receipt were recorded every 1–5 days throughout the flowering period, before flowers started wilting. For each flower, we counted the number of pollinaria removed and noted whether pollen had been received or not. At fruit maturation, we recorded the number of mature fruits produced. For each plant, we estimated male fitness as the total number of removed and deposited pollinaria and female fitness as the product of number of fruits.

Mean values and standard deviations were calculated for each species for all fitness measures using SPSS Release version 17.0.1 and Microsoft Excel 2007 (Microsoft Corporation, Redmond, WA).

Each species were analyzed separately using two‐way ANOVA to compare treatments (the three treatments of manipulation vs. the unmanipulated controls), to examine the percentage of flower manipulated (100%, 50% and 10% vs. the natural condition) and their interaction on proportion of pollinaria removed, proportion of flowers receiving pollinaria, and fruit formation. All proportional data were arcsine square‐root transformed prior to analyses. When the *F* test was significant, means were compared using the Tukey test at 5% error probability.

## 3 RESULTS

Reproductive success in natural conditions of our study species ranged from 29% to 38.3% without differences between examined orchids. In particular, in *S. vomeracea* 38.3% of the flowers had pollinaria removed, 38.3% were pollinated and 32.3% produced fruits; in *S. lingua,* 35.3% of the flowers showed pollinaria removed, 33% received pollinaria, and 29% produced fruits.

**Figure 3:**
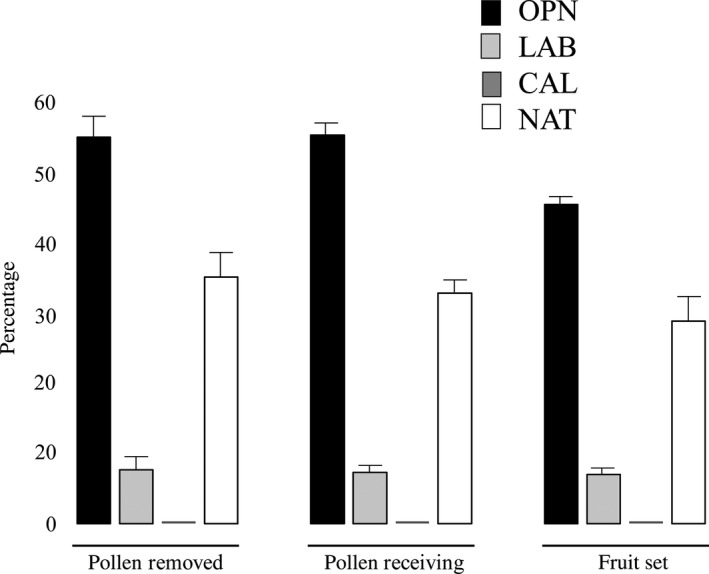
Variation of reproductive success among manipulation, OPN (the petals and sepals have been detached, and the tube shape was opened), LAB (lip was completely removed), and CAL (the flower was open, and the callus surface was painted) and NAT (natural condition) in *Serapias lingua*

Differences among the manipulation groups were highly significant in both species (Tables 1 and 2). In particular, intact flowers of *S. vomeracea* had significantly higher value of pollinaria removed and deposited and fruit set than those with manipulated flowers, while *S. lingua* flowers with the petals and sepals detached and the tube shape opened showed significantly higher value of pollinaria removed and deposited and fruit set than intact flowers (Figure 3).

**Table 1:** ANOVA results of the effects of treatments (OPN, LAB, and CAL), the percentage of flower manipulated (100%, 50%, and 10%), and their interaction on proportion of pollinia removal, proportion of flowers receiving pollinia, and fruit set in *Serapias vomeracea*

Source of variation	*df*	MS	*F*	*p*
Proportion pollinia removal				
Treatments	2	0.82	30.80	<.**001**
% flower manipulated	2	0.21	2.22	.66
Treatments × % flower manipulated	1	0.54	2.12	.34
Error	188			
Proportion of flowers receiving pollinia				
Treatments	2	0.77	29.32	<.**001**
% flower manipulated	2	0.32	3.34	.54
Treatments × % flower manipulated	1	0.25	3.14	.38
Error	188			
Fruit set				
Treatments	2	1.02	22.24	<.**001**
% flower manipulated	2	0.78	0.88	.70
Treatments × % flower manipulated	1	0.42	2.40	.40
Error	188			

Effects with *p* < .05 are shown in bold face type.

**Table 2:** ANOVA results of the effects of treatments (OPN, LAB, and CAL), the percentage of flower manipulated (100%, 50%, and 10%), and their interaction on proportion of pollinia removal, proportion of flowers receiving pollinia, and fruit set in *Serapias lingua*

Source of variation	*df*	MS	*F*	*p*
Proportion pollinia removal				
Treatments	2	1.12	20.70	<.**001**
% flower manipulated	2	0.41	1.22	.60
Treatments × % flower manipulated	1	0.34	2.47	.38
Error	190			
Proportion of flowers receiving pollinia				
Treatments	2	0.87	28.88	<.**001**
% flower manipulated	2	0.44	7.23	.48
Treatments × % flower manipulated	1	0.21	3.26	.27
Error	190			
Fruit set				
Treatments	2	1.24	32.14	<.**001**
% flower manipulated	2	0.18	1.32	.66
Treatments × % flower manipulated	1	0.27	3.16	.22
Error	190			

Effects with *p* < .05 are shown in bold face type.

In detail, in *S. vomeracea,* the three treatments reduce to zero reproductive success both in terms of pollinaria removed/deposited that of fruit set. On the contrary, *S. lingua* plants showed different responses to treatments. Indeed, white painting callus reduce to zero reproductive success, as it happened to *S. vomeracea*. The removal of labellum reduced to a quarter (with only 7.7% of the flowers having pollinaria removed, 7.3% being pollinated, and 7% produced fruits) compared with the control plants. Interestingly, the open flowers, in which the petals and sepals have been detached, showed higher values of reproductive success (with 55.3% of the flowers having pollinaria removed, 55.7% being pollinated, and 45.7% produced fruits) twice higher than the values of plants not subject to manipulation. As the number of pollinaria removed or fruit produced is a result of the visits of insects, our data indicate that the manipulations have effect on the attraction of pollinators.

**Figure 4:**
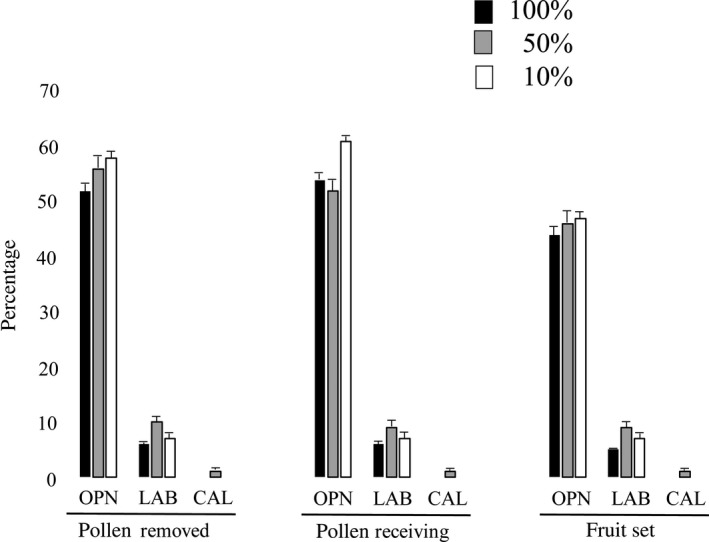
Effects of three manipulation experiments on *Serapias lingua*. OPN (the petals and sepals have been detached, and the tube shape was opened), LAB (lip was completely removed) and CAL (the flower was open, and the callus surface was painted) performed on all flowers (100%), on half flowers (50%), and on 10% of flowers of an inflorescence

There was no significant differences between populations in variation of reproductive success for both examined species (one‐way ANOVA; *F*_3, 388_ = 0.41, *p* = .66, *S. vomeracea*;* F*_3, 389_ = 0.37, *p* = .58, *S. lingua*), Moreover, there were no significant differences when the three treatments were performed on all flowers (‐100), on half flowers (‐50), and on 10% of flowers (‐10) of an inflorescence (Tables 1 and 2, Figure 4).

## 4 DISCUSSION

The percentage of fruits produced by the natural populations of examined species (29%–32.3%) is within the ranges reported for nectarless orchids of temperate zones (Neiland & Wilcock, 1998) and is consistent (just a little higher) with records for previously papers on *Serapias* populations (Bellusci, Pellegrino, Palermo, & Musacchio, 2010; Pellegrino, Bellusci, & Palermo, 2015; Pellegrino, Musacchio, Noce, Palermo, & Widmer, 2005).

The number of deposited/exported pollinaria, likewise the number of fruits produced, depended on the treatment involved. These results indicate that the detached of the petals and sepals, the excision of the labellum, and the painting of callus had effect on pollinaria removal or fruit production and thus on pollinator attraction. Overall, these results suggest that pollinators were largely sensitive to the experimental modification of the flower morphology, which is consistent with the presence of significant selection on individual floral characters.

Previous studies reported for orchids in Mediterranean area that inflorescence size modified male and female reproductive success (Cuartas‐Domínguez & Medel, 2010; Firmage & Cole, 1988).

Modifying the traits of the flowers drastically altered the display of the resulting flower and had a particularly large effect on those parameters that were phenotypically stable among intact flowers. The experimental modifications of the perianth led to a different response of both male and female reproductive success compared with nonmanipulated flowers. In *S. vomeracea* modification of the floral tube significantly reduces reproductive success, suggesting that, to the extent that pollinators preferentially visit intact flowers, one would obtain a decline in visitation. On the contrary, flower modifications of *S. lingua* increase the attraction of insects and thus increase fitness.

These results agree to the different pollination strategy of examined species. Indeed, *S. vomeracea* flowers offer a shelter to insects and the tube‐shaped structure modification has failed to lend to insects the urge to seek refuge inside the flower, which provides a plausible explanation why manipulated flowers of *S. vomeracea* in our study populations had extremely low levels of reproductive success.

Instead, *S. lingua* is a sexually deceptive orchid, and therefore, the opening of the flower made more visible callus (visible at a greater distance) and increased the intensity of scents. Insect males, searching nesting sites or mates, orientate themselves by odor (Ayasse et al., 2000; Schiestl, 2010), but at short distances, when objects subtend a certain visual angle, optical features can be perceived and act as short‐range stimuli (Streinzer, Paulus, & Spaethe, 2009). Thus, the increased male and female reproductive success associated with two of the three trait manipulations can be readily explained by increased attractiveness to pollinators. Pollinators first approach the *S. lingua* flowers guided by the pheromone produced by the flowers, but when close enough, they prefer flowers with open perianth and with the callus exposed.

In the first case, the results highlight that also for an orchid with refuge strategy (such as *S. vomeracea*), visual signals play a key role in pollinator attraction as already demonstrated in rewarding (Duffy & Stout, 2011; Sun, Gross, & Schiestl, 2014; Sun, Schlüter, Gross, & Schiestl, 2015) and food‐deceptive species (Galizia et al., 2005; Jersáková, Jürgens, Šmilauer, & Johnson, 2012; Sletvold & Ågren, 2011). In the second case, our in situ manipulation experiments emphasize the significance of the open perianth, such as callus, as strong visual signals for pollinator attraction and reproductive success in the sexually deceptive species (Peakall et al., 2010; Rakosy et al., 2012; Spaethe, Moser, & Paulus, 2007). These data provide further evidence that the callus form in *S. lingua* is adaptive and thus adds to the olfactory signal to maximize pollinator attraction and reproductive success. This floral trait influences both attractiveness to pollinators and morphological fit between flower and pollinator (Alexandersson & Johnson, 2002). Moreover, the low values (~7%) obtained in experiments in which the labellum was completely removed and the 0% of reproductive success in manipulations in which white painting was applied to the callus surface confirm that both structures help to attract pollinators. But whereas previously it was thought that the pheromone analog produced by the labellum was the principal signal influencing pollinator visitation rate and thus reproductive success, our experiments show that the callus has a key role in attracting insects.

The strong effect of the manipulations on the reproductive success of *Serapias* may be explained by the behavioral patterns and sensory system of its pollinator. In the case of *S. vomeracea* petals, sepals the labellar lobes play a critical role in attracting pollinators. In this species, the labellum not only serves as a landing platform for pollinator insects, but it would serve as a visual indicator of entrance of a refuge or a nest. In the case of open flowers, the insects are not attracted because they do not recognize the possibility of a shelter because they do not identify in this structure a safe place to rest. Conversely, the open flowers of *S. lingua* make more visible the callus that plays a critical role in attracting pollinators in this sexually deceptive orchid.

## CONFLICT OF INTEREST

None declared.

## AUTHOR CONTRIBUTIONS

GP conceived the ideas, designed methodology, and wrote the manuscript; GP and FB collected the data; FB and AMP analyzed the data. All authors contributed critically to the drafts and gave final approval for publication.
